# Foucault's "fearless speech" and the transformation and mentoring of medical students

**DOI:** 10.1186/1747-5341-3-12

**Published:** 2008-04-17

**Authors:** Thomas J Papadimos, Stuart J Murray

**Affiliations:** 1 Department of Anesthesiology, University of Toledo, College of Medicine, 3000 Arlington Avenue, Toledo, Ohio 43614, USA; 2 Department of English, Ryerson University, 350 Victoria Street Toronto, Ontario, M5B 2K3, Canada

## Abstract

In his six 1983 lectures published under the title, *Fearless Speech *(2001), Michel Foucault developed the theme of free speech and its relation to frankness, truth-telling, criticism, and duty. Derived from the ancient Greek word *parrhesia*, Foucault's analysis of free speech is relevant to the mentoring of medical students. This is especially true given the educational and social need to transform future physicians into able citizens who practice a fearless freedom of expression on behalf of their patients, the public, the medical profession, and themselves in the public and political arena. In this paper, we argue that Foucault's understanding of free speech, or *parrhesia*, should be read as an ethical response to the American Medical Association's recent educational effort, *Initiative to Transform Medical Education (ITME): Recommendations for change in the system of medical education *(2007). In this document, the American Medical Association identifies gaps in medical education, emphasizing the need to enhance health system safety and quality, to improve education in training institutions, and to address the inadequacy of physician preparedness in new content areas. These gaps, and their relationship to the ITME goal of promoting excellence in patient care by implementing reform in the US system of medical education, call for a serious consideration and use of Foucault's *parrhesia *in the way that medical students are trained and mentored.

## Introduction

The American Medical Association (AMA) Council on Medical Education has identified three areas of deficiency that need to be addressed: (1) the need to enhance health system safety and quality, (2) the need for enhanced emphasis on education in training institutions, and (3) the inadequacy of physician preparedness in necessary content areas [1]. These areas of concern frame the AMA's recently published *Initiative to Transform Medical Education (ITME): Recommendations for change in the system of medical education*. The explicit goal of this document is to:

Promote excellence in patient care by implementing reform in the medical education and training continuum, from pre-medical preparation and medical school admissions through continuing physician professional development. [1]

More specifically, however, the ITME points to a growing gap between the emerging demands of clinical practice, on the one hand, and the ability of the US system of medical education to meet these demands, on the other. The ITME suggests that the current system of medical education in the US is insufficient in a number of ways. The two main proposed outcomes of the ITME initiative are:

The creation of a system of medical education that better equips young physicians with the knowledge, skills, attitudes, and values necessary to provide quality medical care and the ability to continually update their learning; and the availability of appropriate resources, including funding, faculty, clinical sites, and technology to support needed changes in medical education across the continuum. [1]

The report then makes ten recommendations in support of these outcomes. This essay will respond in detail to three of these recommendations (Nos. 5, 6, and 7) insofar as they are relevant to the topic of medical mentorship in university and clinical settings. They are as follows:

(1) Ensure that faculty at all stages of the educational continuum are prepared to teach new content, employ new methods of teaching and evaluation, and act as role models for learners.

(2) Ensure that the organizational environment in medical schools and teaching hospitals tangibly values and rewards participation in education.

(3) Ensure that the learning environment throughout the medical education continuum is conducive to the development of appropriate attitudes, behaviors and values, as well as knowledge and skills. [1]

These three recommendations are, in themselves, a tall order. How, we might ask, can those who teach medicine best ensure that they are apt role models, that they demonstrably value and reward meaningful participation in the educational process, and more significantly, ensure the transfer not just of clinical knowledge and skills, but foremost, the attitudes, behaviors, and values appropriate to a caring and responsible physician? With recommendations such as these, clearly the AMA is gesturing to a kind of medical education that reaches beyond the instruction of knowledge, skills, and information. Those who teach medicine therefore have a dual responsibility to society: they ought to provide society both with (1) competent medical practitioners, and (2) able citizens. In other words, medical faculty should teach students not only to be capable practitioners of their art, but also to be thoughtful, questioning professionals in regard to the society around them.

How shall we best address these ITME challenges? This question is all the more exigent because the physician stands at the crossroads of power, knowledge, and technology. And in today's society, knowledge and technology are scarcely separable from power-relations and their effects [2]. Here, the earlier work of Foucault is helpful in understanding power. For him, power is not a "thing," it is not a possession that can be wielded and deployed; instead, we find ourselves within a scientific and technological power matrix, as nodes or circuits in the perpetual negotiation of power, politics, and knowledge. In this sense, medical knowledge is a form of what Foucault called "biopower," the power over the life of the biological body. Foucault notes a proliferation of medical techniques beginning in the eighteenth century, an important historical development because medical knowledge was able to avert some of the imminent risks of disease and death. Foucault writes: "Power would no longer be dealing simply with legal subjects over whom the ultimate dominion was death, but with living beings, and the mastery it would be able to exercise over them would have to be applied at the level of life itself" [3]. This made medicine a social and political concern. "For the first time in history, no doubt, biological existence was reflected in political existence" [3]. On the one hand, biopower's control over life was effected at the level of the individual (through what Foucault calls "anatomo-politics"), while on the other, biopower became concerned with the administration and regulation of biological bodies insofar as they comprise populations (through what Foucault calls "bio-politics"). The goal of biopolitics was – and still is – "to rationalize the problems presented to governmental practice by the phenomena characteristic of a group of living human beings constituted as a population: [through] health, sanitation, birthrate, longevity, race..." [4]. Because the physician occupies a privileged place with respect to power/knowledge, technologies, and populations, appropriately mentoring medical students is a crucial part of their social and ethical education. Medical students must become able citizens who not only possess the critical skills necessary to understand how power/knowledge operates, but they must develop the capacity to expose and to challenge this power, when required – to speak out fearlessly on behalf of their patients, their profession, themselves, and society in general.

In sum, medical students must learn to practice *parrhesia*, they must speak fearlessly. This does not exactly mean that they will speak without fear; rather, it means that they will learn to have the courage to speak under fearful circumstances – to address and to critique those institutions or individuals who control more power, knowledge, and technology than the one who speaks. It means "speaking truth to power," as Foucault has said. Such an attitude, behavior, or value cannot exactly be "taught" as a skill or as piece of positive knowledge. It calls for an apprenticeship by mentors who will foster such an ethos in their students, who demonstrate *parrhesia *themselves, and who actively encourage new discourses in their teaching, their research, and beyond. Such a transformation would begin to address the ITME's challenges, even if it necessitates a critique of the US system of medical education and of the AMA itself.

## Discussion

### What is *parrhesia*?

*Parrhesia *means free speech, but it is more than this: it is also the duty to speak in a situation in which one is not altogether "free" to do so. It is to speak in a situation in which one's speech carries a certain risk to one's reputation or even to one's life. Foucault writes:

*Parrhesia*... is linked to courage in the face of danger: it demands the courage to speak the truth in spite of some danger.... When you accept the parrhesiastic game in which your own life is exposed, you are taking up a specific relationship to yourself: you risk death to tell the truth instead of reposing in the security of a life where the truth goes unspoken.... *Parrhesia *is a form of criticism, either towards another or towards oneself, but always in a situation where the speaker or confessor is in a position of inferiority with respect to the interlocutor. The *parrhesiastes *is always less powerful than the one with whom he speaks. [5]

We shall refer to this definition throughout the essay. However, in a nutshell we might say that, in order for speech to qualify as *parrhesia*, certain conditions must be met – speaking out in a social situation that places the speaker in danger or at risk because there is an imbalance of power or status between the speaker and his or her audience. Moreover, in the *parrhesiastic *situation, the audience does not want to hear the speech because it contains a deep criticism or critique of the current order of things, for which those in power (the audience) are somehow responsible. It forces those in power to account for themselves and their actions. It therefore takes great courage to speak out, and yet despite the risks, the speaker experiences a social and political duty to speak all the same.

The concept of *parrhesia *helps us to contextualize the "attitudes," "behaviors," and "values" mentioned in the ITME recommendations above. As Foucault notes, the *parrhesiastes *– the one who speaks with *parrhesia *– is engaged in an ethical decision. *Parrhesia *is a self-relation. For this reason, *parrhesia *can also be understood as the kind of speech I have with myself when I plainly tell myself things I do not really want to hear, when I am faced with an agonizing decision, and I find the courage to face my fears, my uncertainty, and to ask myself whether I am really speaking or acting ethically. I risk my self in order to be myself, authentically [6].

We might rightly ask how we know with certainty that the one who speaks with *parrhesia *is a truth-teller. How do we know the he or she possesses the truth? The modern scientist (most of our readers) will logically demand some sort of "evidence" – something that will be verifiable according to the wisdom of the scientific method. This led Foucault to suggest: "It appears that *parrhesia*, in this Greek sense, can no longer occur in our modern epistemological framework" [5]. The self-relation of the *parrhesiastes *is, after all, first an ethical and spiritual relation, not explicitly a relation of knowledge. But modern knowledge is a relatively recent invention, and Foucault takes pains to explain modern epistemology as the gradual ascendency of *mental *evidence beginning roughly with Descartes in the seventeenth century. According to Descartes, "evidence" is given to consciousness without any possible doubt when it is "clear and distinct" (*omne illus verum est, quod clare et distincte percipitur*). Since Descartes, philosophical and scientific thought (the two were not yet separate) asks the following question, according to Foucault: "what... enables the subject to have access to the truth?" [7]. The answer, if we rely on our modern epistemological framework, is the following: "it is assumed that what gives access to the truth, the condition for the subject's access to the truth, is knowledge (*connaissance*) and knowledge alone" [7]. The modern presumption here is that true knowledge is mental, that "evidence" is pure – and that true knowledge means disinterested scientific research, ostensibly free from the body, from the emotions, and from any other "special interests," or – to use another modern word – from the myriad *stakeholders *in the production of "truth."

Foucault's critical response to our "modern epistemological framework" is twofold. First, he exposes the self-deceptive error of modern epistemology, that it could be disinterested and free. And second, he suggests that the concept of *parrhesia *would allow us to adopt and adapt an ancient Greek wisdom that points to the *ethical *and *spiritual *dimensions of the pursuit of knowledge, thus proposing a model that would acknowledge the ruses of modern epistemology while allowing us to move beyond them. First, then, we must accept Foucault's insights concerning power/knowledge, that knowledge is never free of power. Foucault writes: "Truth is not out there waiting to be discovered, it is created in the interest of those who exert the most power" [8]. In the case of medicine, certainly in the US and also in large parts of the present-day world, this is obvious when we consider the influence of financial rewards, our careers and promotion, government agencies that regulate and monitor and sponsor knowledge-production and knowledge-transfer, the myriad interests of public policy decision-makers, the pharmaceutical industry, the insurance industry, various government lobbies, the legal-juridical complex, the convergence of research and business interests – and the list goes on and on. Suffice it to say that this is only the barest allusion to some ways that power and knowledge are imbricated in the scientific enterprise, pointing beyond the medical continuum to the real gatekeepers of scientific knowledge or "truth." It is for this reason that Foucault speaks of a "regime of truth" [2]. Given this nexus of power, knowledge, and technology, it seems only just that we should find ways to challenge those who wield such power, to expose corruption, and to speak out. Rather than presume a modern epistemological framework, and act as if we could speak disinterestedly in the name of a true knowledge that would itself be free from the vagaries of power, *parrhesia *would be a kind of speech that acknowledges this power and wrestles with it. The *parrhesiastes *speaks from within the situation, and does not pretend to occupy a space that is epistemologically neutral and free from constraint.

The ethical and spiritual self-relation of the *parrhesiastes *thus represents a compelling critique of the modern epistemological framework. And in a world ruled by modern epistemology, such speech itself must be *parrhesiastic*! Foucault returns us to the ancient Greeks not out of some philosophical nostalgia; nor does he wish for a wholesale return to a time long gone. Rather, taking *parrhesia *seriously raises a number of difficult questions for our own ways of knowing. Can an ethical and spiritual self-relation still speak to us? What is *parrhesia *or free speech if it is an ethical and spiritual relation, an agonistic relation? Against the "modern epistemological framework" of philosophy and science, Foucault argues that spirituality is "the search, the practice, and experience through which the subject carries out the necessary transformations on himself in order to have access to the truth" [7]. Here we must stress the transformation of the individual, which might also be conceived as a kind of conversion. This is a radical reversal of modern epistemology. In modern epistemology, we are presumed to have access to the truth; here, in contradistinction, in order to have access to the truth, one must struggle to stand in the correct spiritual and ethical relation, one must be transformed. It is not enough to have *mental *evidence, but the condition of possibility for access to true knowledge is the fruit of a kind of spiritual and ethical apprenticeship. This apprenticeship prepares the way for knowledge, and allows the individual to transform evidence and information into meaningful knowledge and truth. Foucault writes: "In short, in truth and in access to the truth, there is something that fulfills the subject himself, which fulfills or transfigures his very being. In short, I think we can say that in and of itself an act of knowledge could never give access to the truth unless it was prepared, accompanied, doubled, and completed by a certain transformation of the subject" [7].

### Transformation in medical schools

Medical schools, simply put, are places where teachers and students engage in clinical work and research. Patients must be cared for, students must be taught, faculty must publish (usually), and young, competent physicians, who score well on their licensing examinations and capture seats in "outstanding" residency training programs, must be produced. Producing such a fine product (a premiere medical student), in addition to quality research, allows a medical school to more effectively compete against other institutions for grant monies and well-recognized faculty candidates through an enhanced reputation. Against this backdrop, in the US, the legal profession (through malpractice) and the insurance industry/government axis (through curtailment of remuneration and closer regulation) have allowed faculties of medicine less latitude in what students and residents can do vis-à-vis the patient. Increasingly, faculty members are concerned that the American system of medical education must now also teach due diligence, and must develop an adequate response if we hope to graduate self-sufficient practitioners (at least in their initial practice of medicine) [9,10]. Furthermore, in the US, students enter medical practice with a burden of debt that causes them so much anxiety that they are often directed toward specialties or practices that are more lucrative, even though as students they may have preferred to do something else [11]. This is neither good for the physician nor for the public at large. US medical education has been *transformed *over the past four decades by public *discourses *initiated by the legal community, government, insurance industry, pharmaceutical firms, patients, and even medical faculties themselves. The power wielded by the media, government, corporations, patient advocacy groups, etc., is real. These *transformations *over recent decades have produced a *truth *about US medical education that differs from the *truth *of 40 years ago. The ITME acknowledges some of the challenges wrought by these transformations. But how should we respond?

We argued above that medicine is at the heart of a power/knowledge and technology complex. Medical practice involves *power *(who hires you, who pays you, who sues you, which hospital gives you privileges and to what extent), *knowledge *(board certification, locating a practice, how to administer a practice, how patients are billed, how procedures are done), and *technology *(equipment, procedures, laboratory testing, and DNA counseling). Foucault is useful for a discussion of medical education because he demonstrates that current practices – while they appear to be "neutral" or obviously "true" to us – nevertheless rely on social, historical, and political contingencies. Foucault promised a "history of the present" in his work. In other words, when we understand how things came to be as they are, now, we recognize that they might have been otherwise; this is empowering, because it allows us to imagine how things *could be *otherwise, how they *could be *transformed, in the future. He writes:

The political and social processes by which Western European societies were put in order are not very apparent, have been forgotten, or have become habitual. They are part of our most familiar landscape, and we don't perceive them anymore. But most of them once scandalized people. It is one of my targets to show people that a lot of things that are part of their landscape – that people think are universal – are the result of some very precise historical changes. All my analyses are against the idea of institutions and show which space of freedom we can still enjoy and how many changes can still be made [12].

With respect to the US system of medical education, we have grown too used to the status quo, too used to accepting as truth what is only a habit. We must try to imagine how things could be otherwise.

Imparting *parrhesiastic *attitudes, behaviors, and values in medical students is one way to give them the capacity to challenge the status quo, to transform medical education with an eye to the future, and to offer something practical in our present moment. We have argued that medical students must transform themselves into able citizens – not just skilled technicians – and that this necessitates the use of fearless speech throughout their careers. However, students often cannot positively transform themselves before they enter into practice because their experience and knowledge is limited in this respect; moreover, the demands and seductions of burgeoning technologies and the nexus of power/knowledge makes authentic self-transformation a Herculean task for the best of us, let alone the uninitiated. If medical students are "released into the wild" (medical practice) without guidance and experience in regard to *parrhesia *they will not only be less able citizens, but disabled citizens in regard to their ability to be proactive, or even reactive, when confronted with issues of significance concerning themselves, their patients, government, business, or society at large. In this effort, effective mentors and role models are vital. But the prospect of transforming medical education through such mentorship seems gloomy. While US medical faculties have increased their numbers by 600% in the latter part of the twentieth century, mentoring has deteriorated [13]. Part of the difficulty has been that, while mentoring has been identified as a critical step to success, too few medical students and educators recognize its value [14].

Moreover, as most modern systems of medical education are increasingly governed by measurable "outcomes," "deliverables," and "evidence-based" agendas, along with a corporate vocabulary of "key performance indicators," "best practices," and the like, it is difficult to imagine how a discourse on "attitudes," "behaviors," and "values" could gain a foothold – let alone any discourse on "spirituality"! – since these cannot be measured within our modern epistemological framework. How would such mentorship be implemented, counted, regulated, and monitored? Who would be responsible, and according to what – or whose – terms and standards? Is it not time to have this discussion? Certainly, medical apprenticeship or mentorship cannot be conceived as the transfer of some positive piece of knowledge or routinely executable skill. In this regard, Foucault speaks of three types of "mastership," which we might also translate as mentorship or apprenticeship: (1) "the mastership of competence," where "knowledge, principles, abilities, know-how and so on" are passed along. This first type of mastership speaks more to the transfer of positive knowledge and skills, while the next two relate directly to *parrhesia*: (2) "Mastership through example," where a "model of behavior" is passed on, or even a tradition (which, etymologically, means "to hand down"); and (3) "the Socratic mastership of dilemma and discovery practiced through dialogue" [7]. Socratic mastership is perhaps closest to what we mean by *parrhesia *here, for Socrates is the quintessential *parrhesiastes*, willing to go to his death to speak the truth. Socrates teaches the student "that he does not know and, at the same time, that he knows more than he thinks he does" [7]. While this seems cryptic, the message is simple: the limits of the student's knowledge – in our sense, the limits of a modern epistemological framework – must be acknowledged, but this acknowledgement is a form of knowledge and empowerment in itself. After all, Socratic wisdom is captured in the adage, "I know that I know nothing." Once we admit our limitations, we are on the road to true knowledge.

More importantly, however, Socrates teaches the student not just how to respect the limits of his knowledge, but he teaches the student *how *to relate to himself, how to care for himself – a relation that is both a kind of practice or exercise (*askesis*) and love (*eros*) [7]. Recall that this spiritual and ethical relation is the condition of possibility of true knowledge for the *parrhesiastes*. If we apply this in the context of a medical education, while medical students learn facts and knowledge in medical school, knowing is not thinking. Students must evolve into thinkers who also learn to use *parrhesia *as a "practice of speaking the truth which addresses, not only the city, but the soul, the psyche, of the individual" [15]. This, in turn, means that the good mentor teaches the pupil how to be a good teacher. At the same time, while the mentor can never be a *parrhesiastes *vis-à-vis the student because the mentor is in a position of power and authority over the student (and is not the one at risk), like Socrates, the mentor must nevertheless publicly exercise *parrhesia*, when it is called for, in the face of *his or her *superiors. While mentors may not be able to inculcate particular moral qualities in their students, in the very least, acting as a role model opens the possibility for a *parrhesiastic *relation by demonstrating and embodying sincerity in their opinions and courage in their actions.

The good mentor demonstrates and embodies the critical attitudes, behaviors, and values of the *parrhesiates*, but more than this, she or he allows the student to practice *parrhesia *by cultivating a non-hostile environment, a "space of appearance" [16], to exist where she or he can be freely questioned by the student. In providing such latitude to the student, the mentor not only allows the student to learn to challenge through frank interrogation, but at the same time the mentor will be preparing the student to appear in society's public arena to challenge people, laws, institutions, and government on behalf of patients, the public's health, and the medical profession itself. Fostering fearless speech will not only transform educational systems, but will transform the individual student into a subject who has access to the truth and who conducts him- or herself more ethically vis-à-vis other individuals and institutions.

Foucault makes it abundantly clear that *parrhesia *is "played" between a truth-teller and an "interlocutor" [5]. In relation to mentorship in medical schools, this means between a faculty member and his or her medical students. Because *parrhesia *involves a risk, the danger is that the speaker tells the interlocutor the truth – a criticism that might cause anger or perhaps even call for disciplinary action. As mentioned above, this criticism could be of the interlocutor, the institution, or a criticism of the *parrhesiastes *(the student) him- or herself. In this latter case, speakers might be confessing something they have done wrong. This characteristic is important to cultivate in medical students. If they can critique their mentors and admit their failings (without being severely chastised), then eventually they will acquire the ability to criticize in the public arena and, moreover, receive criticism in return.

Finally, because the function of *parrhesia*, or fearless speech, is criticism, and that such frankness, or truth-telling, can be a danger to the speaker, we come to an inevitable conclusion: *parrhesia *is a *duty *[5]. How so? The speaker could keep silent, could lie, flatter, or try persuasion. But this is not the course of a *parrhesiastes *[5]. Here Foucault distinguishes between the use of rhetoric for persuasion and the *parrhesiastic *desire for a direct kind of honest speech that is non-rhetorical, unembellished. The *parrhesiastes *has chosen "a specific relation to moral laws through freedom and duty" [5]. He or she has decided that telling the truth will help a person, people, or a situation. The duty in this situation is an obligation to articulate with clarity: "That is, *parrhesia *reveals to the listener the listener's own truth, the listener's ethos, by speaking in such a way that the listener is thrown back upon himself" [15]. While the individual is free to refuse to speak, the *parrhesiastes *experiences such speech as an imperative, as ethically necessary and unavoidable. Bravely addressing one's superiors, challenging power and authority, is a capacity we need in tomorrow's physicians.

### Fearless speech and the specific intellectual

In the past, this ability to address power and authority on behalf of one's society was demonstrated by individuals who were considered "universal intellectuals," i.e., those public figures at large who wrote for, or who otherwise expressed, the conscience of society [2]. In Foucault's words: "the intellectual *par excellence *used to be the writer: as a universal consciousness, a free subject, he was counterposed to those intellectuals who were merely *competent instances *in the service of the State or Capital – technicians, magistrates, teachers" [2]. However, Foucault claims that the "universal intellectual" no longer exists; the classical left-leaning intellectual, or "spokesman of the universal," no longer serves as the conscience of society. Instead, this kind of intellectual has been displaced by the "specific intellectual" who works "competently" within specific sectors of society, "at the precise points where their own conditions of life or work situate them (housing, the hospital, the asylum, the laboratory, the university, family and sexual relations)" [2]. Specific intellectuals therefore have a contingent attachment to their profession, rather than an essential and universal attachment to society. Specific intellectuals are experts in a narrow field or subject matter, concerning themselves with "specific" problems that are "non-universal." We might find in this definition a way to characterize the physician, who is encouraged to further "specialize" in a subfield or particular branch of medicine.

The problem, as Foucault sees it, is that the specific intellectual is too narrowly concerned – and lacks the means – to address power and authority. And yet, the need for this is greater than ever. Today, many technical experts, such as physicians, sociologists, scientists, judges, and attorneys are working in their own distinct fields: "This process explains how, even as the writer tends to disappear as a figurehead, the university and the academic emerge, if not as principle elements, at least as 'exchangers', privileged points of intersection" [2]. The writer of public conscience has all but disappeared, and we see the rise of an intellectual class, of "specification," and an increasing collusion between power, knowledge, and technology. The university is home to these specialists, occupying individual silos where knowledge is produced and consumed for tiny segments of the population. As Foucault writes, the "specific intellectual" is:

no longer he who bears the values of all, [he who] opposes the unjust sovereign or his ministers and makes his cry resound beyond the grave. It is rather he [the specific intellectual] who, along with a handful of others, has at his disposal, whether in the service of the State or against it, powers which can either benefit or irrevocably destroy life. He is no longer the rhapsodist of the eternal, but the strategist of life and death. [2]

We might say that the intellectual – increasingly only the "specific intellectual" – has become an unwitting agent, a node, a circuit in the exercise of biopolitical State power. Specific intellectuals work in the service of biopower, and are increasingly unable to levy a critique because the landscape is so familiar and our habits too ingrained. Foucault wrote these words nearly a generation ago and they seem to have come to pass. It is at this juncture where readers may detect a warning in Foucault's narrative, as if he himself were an anachronism (the one who bears the values of all and opposes the unjust), no longer comprehensible – or just barely? – in a world governed by specific intellectualism, measurable "outcomes," "deliverables," and "evidence-based" agendas, along with a corporate vocabulary of "key performance indicators," "best practices," etc. Perhaps it is time for the ethics of fearless speech, to transform specific intellectuals as those who – like the physicians of tomorrow – will find themselves positioned at the "privileged points of intersection," in the interstices of power, knowledge, and technology.

The physicians of tomorrow will have an ever-greater political responsibility, following in the footsteps of people such as Oppenheimer, a scientist who specifically understood the nuclear age. About sixty years ago, Foucault writes, for the first time "the intellectual was hounded by political powers, no longer on account of a general discourse which he conducted, but because of the knowledge at his disposal: it was at this level that he constituted a political threat" [2]. How will our future physicians navigate these waters? Here, under the pressure of economic and political conditions (if these have not yet merged entirely), is where fearless speech will be required. Patients, electorates, politicians, friends and foes alike will need to be addressed by physicians and bioethicists with frank criticism, and truth, through an obligation of duty, about problems and discourses that smolder, or even rage, throughout society. Cloning, abortion, healthcare insurance, healthcare access, pharmaceuticals, genetic engineering, malpractice, war, famine, and politics are engulfing the physician as specific intellectual. We need fearless speech if we hope to develop a vocabulary that will be adequate to the ethical challenges wrought by our technologies. Fearless speech in the physician as a specific intellectual is important especially in relations to political and bureaucratic structures. Mentors should not only teach medical students to practice *parrhesia*, but they should instruct medical students that they can learn much by allowing others (nurses, allied health care professionals, and patients) to speak freely towards them, too, especially when they become attending physicians. In this way, they will begin to transform the meaning of themselves as "specific intellectuals."

An excellent example of fearless speech on the part of medical students occurred at the University of Toledo College of Medicine recently. In the autumn of 2006, the medical student body embarked on an effort to limit pharmaceutical company gifts and meals to faculty, residents, and students on the Health Science Campus. This position was opposed by a number of faculty members, administrators, and residents. However, the students were supported by many faculty members, and by a history of similar initiatives at other universities, as well as by the medical literature. Through the use of fearless speech in heated debates (both public and private), the students were able to initiate, define, support, and dominate this discourse. In practicing *parrhesia*, the students were truth-tellers who successfully criticized the position of the status quo held by some of the faculty, administrators, and residents (interlocutors), and within six months they ultimately, and overwhelmingly, prevailed.

## Conclusion: ITME, Foucault, and fearless speech

Those who represent the power/knowledge base and have the ability to influence discourses within medical communities include broad groups that have been identified through the ITME process: practicing physicians; medical educators and medical management organizations; payers and purchasers; accreditation, certification and licensure organizations; and other health professionals (including those in public health) [1]. The broad array of stakeholders involved in this process may have differing opinions as to the discourses necessary to effect change, or as to whether any changes truly need to be effected. In the words of the AMA's ITME: "Successful reform also requires attention to factors that influence the educational process, including faculty rewards systems, the attitudes and values displayed by supervisors and peers as part of the learning environment, and the financing of medical education and health care" [1].

We began this essay as a response to three of the ITME's recommendations for reform to the US system of medical education. We framed the ITME recommendations as a question of medical mentorship in the educational setting: Could we imagine how new content might be taught, how best to act as role models for learners, how to tangibly value and reward participation in education, and how to foster the appropriate attitudes, behaviors, and values, as well as knowledge and skills? We answered that "implementing" these reforms will be no simple task. We have called for a *parrhesiastic *form of mentorship that cultivates able citizens who are schooled in the art of critique, and who are taught the spiritual and ethical value of speaking out fearlessly in the face of power and authority.

Certainly we do not expect consensus on the ills of the US system of medical education nor on the best prescription for its improvement. We have argued, instead, that Foucault's concept of *parrhesia *should be a crucial part of medical mentorship. In their training as "specific intellectuals," medical students need more than the ability to formulate a differential diagnosis. They must acquire the capacity to pursue *parrhesiastic *truth-telling as an activity, an "art of life (*techne tou biou*)" [5]. This philosophical problem of the twenty-first century is the same problem Socrates and other ancient philosophers identified in the fifth century BC: "who is able to tell the truth, about what, with what consequences, and with what relation to power" [5]? Medical students not only need good mentoring to successfully transition into medical practice as able practitioners, they also need an apprenticeship in transformation, to become able citizens. As able citizens who wield fearless speech, our future physicians – today's medical students – will be best equipped to influence the discourses in society that contribute to the creation of truth, and to take their place as critical intellectuals who can face "what is" while speaking and working fearlessly toward "what can be."

## Competing interests

The authors declare they have no competing interests.

## Authors' contributions

TJP and SJM were responsible for the entire manuscript.

## About the authors

Thomas J. Papadimos is an Associate Professor of Anesthesiology and Medical Microbiology and Immunology at the University of Toledo, College of Medicine, in Toledo, Ohio USA, and Adjunct Clinical Associate Professor of Anesthesiology at the University of Michigan, School of Medicine, Ann Arbor, Michigan USA. He is interested in medical student/resident education and mentoring, and the moral obligation of physicians to provide healthcare to medical outliers and impoverished populations.

Stuart J. Murray is Assistant Professor of Rhetoric and Writing in the Department of English at Ryerson University, Toronto, Canada. Trained in philosophy and rhetorical theory and criticism, his research interests include ethics, biopolitics, and subjectivity. His collection, *Critical Interventions in the Ethics of Healthcare*, edited with Dave Holmes, is forthcoming with Ashgate Publishing.
